# Arterial Stiffness Assessment Using the Arteriograph in Patients with Moderate–Severe OSA and Metabolic Syndrome—A Pilot Study

**DOI:** 10.3390/jcm10184238

**Published:** 2021-09-18

**Authors:** Ioana Mădălina Zota, Cristian Stătescu, Radu Andy Sascău, Mihai Roca, Larisa Anghel, Ovidiu Mitu, Cristina Mihaela Ghiciuc, Daniela Boisteanu, Razvan Anghel, Sebastian Romica Cozma, Lucia Corina Dima-Cozma, Florin Mitu

**Affiliations:** 1 Department of Medical Specialties (I), Faculty of Medicine, Grigore T. Popa University of Medicine and Pharmacy Iași, 700115 Iasi, Romania; ioana-madalina.chiorescu@umfiasi.ro (I.M.Z.); cristian.statescu@umfiasi.ro (C.S.); radu.sascau@umfiasi.ro (R.A.S.); mihai.c.roca@umfiasi.ro (M.R.); larisa.anghel@umfiasi.ro (L.A.); ovidiu.mitu@umfiasi.ro (O.M.); razvan.anghel@umfiasi.ro (R.A.); cozma.dima@umfiasi.ro (L.C.D.-C.); florin.mitu@umfiasi.ro (F.M.); 2 Department of Morpho-Functional Sciences (II), Faculty of Medicine, Grigore T. Popa University of Medicine and Pharmacy Iași, 700115 Iasi, Romania; 3 Department of Medical Specialties (III), Faculty of Medicine, Grigore T. Popa University of Medicine and Pharmacy Iași, 700115 Iasi, Romania; daniela.boisteanu@umfiasi.ro; 4 Department of Surgery (II), Faculty of Medicine, Grigore T. Popa University of Medicine and Pharmacy Iași, 700115 Iasi, Romania; sebastian.cozma@umfiasi.ro

**Keywords:** arterial stiffness, Arteriograph, obstructive sleep apnea (OSA), continuous positive airway pressure (CPAP), metabolic syndrome (MS), adherence

## Abstract

Background: Both obstructive sleep apnea (OSA) and metabolic syndrome (MS) promote arterial stiffening. As a basis for this study, we presumed that arterial stiffness could be assessed using the Arteriograph (TensioMed, Budapest, Hungary) to detect early modifications induced by continuous positive airway therapy (CPAP) in reversing this detrimental vascular remodeling. Arterial stiffness is increasingly acknowledged as a major cardiovascular risk factor and a marker of subclinical hypertension-mediated organ damage. The aim of this pilot study was to evaluate the arterial stiffness changes in patients with moderate–severe OSA and MS after short-term CPAP use. Methods: We performed a prospective study that included patients with moderate–severe OSA and MS who had not undergone previous CPAP therapy. All subjects underwent clinical examination and arterial stiffness assessment using the oscillometric technique with Arteriograph (TensioMed, Budapest, Hungary) detection before and after 8-week CPAP therapy. Results: 39 patients with moderate–severe OSA were included. Eight weeks of CPAP therapy significantly improved central systolic blood pressure (Δ = −11.4 mmHg, *p* = 0.009), aortic pulse wave velocity (aoPWV: Δ = −0.66 m/s, *p* = 0.03), and aortic augmentation index (aoAix: Δ = −8.25%, *p* = 0.01) only in patients who used the device for a minimum of 4 h/night (*n* = 20). Conclusions: Arterial stiffness was improved only among CPAP adherent patients and could be detected using the Arteriograph (TensioMed, Budapest, Hungary), which involves a noninvasive procedure that is easy to implement for the clinical evaluation of arterial stiffness.

## 1. Introduction

Aging and cardiometabolic disease generates morphological and functional changes in the arterial wall, inducing endothelial dysfunction of the small vessels and stiffening of the larger arteries. The reduced compliance of the arterial wall leads to increased pulse wave travel velocities (PWV) and to an earlier reflection of the systolic pulse wave from peripheral bifurcation points [1]. PWV, the most direct measure of arterial stiffness [2], is generally associated with cardiovascular mortality in general and with all-cause mortality among subjects with arterial hypertension [3]. Carotid–femoral pulse wave velocity PWV (cfPWV) [4] is the reference technique for arterial stiffness assessment; however, measurements are time consuming and difficult to implement in routine clinical practice [5], resulting in arterial stiffness assessment being almost exclusively a research activity [4]. Consequently, the results using an oscillometric technique (Arteriograph, TensioMed, Budapest, Hungary) have been validated compared to an invasive method (cardiac catheterization) [5,6]. Studies that have compared the results using the Arteriograph (TensioMed, Budapest, Hungary) to those obtained using the standard arterial stiffness assessment methods showed similar Aix (augmentation index) and PWV (pulse wave velocity) values despite the fact that the techniques are not interchangeable [5,7,8]. The Arteriograph (TensioMed, Budapest, Hungary) calculates central (aortic) pulse wave velocity (aoPWV), aortic augmentation (aoAix), and central (aortic) systolic blood pressure (aoSBP). PWV and Aix are both cardiovascular risk factor predictors [5,9,10]. However, while PWV reflects only the studied arterial segment, Aix is influenced by the characteristics of the entire arterial system that participates in pulse wave reflection [11].

Obstructive sleep apnea (OSA) is defined as a form of sleep-disordered breathing in which repetitive collapse of the upper respiratory airways induces iterative episodes of apnea and hypopnea, leading to micro-awakenings, fragmented sleep, depression, and a poor quality of life [12,13]. OSA is associated with altered intrathoracic pressure balance, overactivation of the sympathetic nervous system, and renin-angiotensin systems, along with pro-inflammatory status and oxidative stress [12,14,15,16], which promote and aggravate arterial stiffening [11,17], especially in patients with associated high blood pressure (HBP) or metabolic syndrome (MS) [11]. The impact of continuous positive airway pressure (CPAP) therapy on arterial stiffening in OSA patients remains conflicting [12,14,18,19,20], being dependent on OSA severity [21], daytime sleepiness [22], patient comorbidities [14,19], and CPAP adherence [21]. Several reports have shown a significant decrease in PWV after 1–6 months of CPAP therapy [20,23,24,25]. Information regarding the long-term effect of CPAP is scarce, with a previous study [26] showing that PWV decreases over the first 6 months before gradually increasing from 6 to 24 months (without exceeding baseline values) [23].

Metabolic syndrome is frequently associated with OSA [27,28], but it is also associated with an accelerated progression of arterial stiffening [29]. CPAP increases the chance of reversing arterial stiffening in patients with moderate–severe OSA [30], but Garleneau et al. [31] reported that arterial stiffening progression in obese OSA patients at 5-year follow-up was not influenced by CPAP adherence. Furthermore, a previous meta-analysis found that the proportion of adherent patients does not impact the benefit of CPAP on arterial stiffness [18].

There are debates on whether arterial stiffness improves under CPAP therapy in OSA patients with MS. The purpose of this pilot study was to use oscillometry (via Arteriograph (TensioMed, Budapest, Hungary) detection) to verify if there are differences in the progression of arterial stiffness in moderate–severe OSA patients with MS after receiving short-term (8 weeks) CPAP therapy. Second, we analyzed the relationship between OSA severity and arterial stiffness parameters. 

## 2. Materials and Methods

### 2.1. Patients

Clinically stable patients with newly diagnosed moderate or severe OSA and MS, were prospectively recruited in the IIIrd Pneumology Clinic in Iași from January to December 2018, prior to the initiation of CPAP therapy. Moderate or severe OSA was diagnosed as having apnea–hypopnea Index (AHI) values of 15–30 events/h and >30 events/h, respectively. MS was diagnosed according to the American Heart Association/National Heart, Lung, and Blood Institute updated National Cholesterol Education Program—Adult Treatment Panel III criteria [32], which requires the presence of at least three of the following factors: fasting glucose ≥ 100 mg/dL or current treatment for diabetes, high blood pressure or current blood pressure-lowering treatment, abdominal obesity (waist circumference ≥ 102 cm for males and ≥ 88 cm for females), hypertriglyceridemia (TG levels > 150 mg/dL or current treatment for hypertriglyceridemia), high-density lipoprotein (HDL) cholesterol < 40 mg/dL for males and < 50 mg/dL for females or current treatment with statins. The 2018 European guidelines for the management of hypertension specifically recommend the use of ambulatory blood pressure monitoring (ABPM) in OSA patients [4]. As such, we defined high blood pressure as mean 24 h blood pressure (BP) ≥ 130/80 mmHg, mean daytime BP ≥ 135/85 mmHg, or mean nighttime BP ≥ 120/70 mmHg [4]. An OSA diagnosis was established by ambulatory or in-hospital six-channel cardiorespiratory polygraphy, using either a Philips Respironics Alice Night One or a DeVilbiss Porti 7 device. The recordings were manually scored by experienced sleep physiologists, according to the third International Classification of Sleep Disorders criteria [33]. Apnea was defined as a reduction in oro-nasal airflow by ≥90% for at least 10 s. Hypopnea was defined as a reduction in oro-nasal airflow by ≥30% for at least 10 s, that is associated with a ≥3% decrease in peripheral oxygen saturation. CPAP effective pressure autotitration in the sleep laboratory was determined using DreamStation Auto CPAP (Philips Respironics, Murrysville, PA, USA), REMstar Auto C-Flex CPAP (Philips Respironics, Murrysville, PA, USA), or a AirSense 10 Autoset CPAP (ResMed, San Diego, CA, USA). Follow-up cardiorespiratory polygraphy data were not collected due to the short follow-up of patients (8 weeks). Daytime sleepiness was assessed using the standard Epworth questionnaire at baseline and after 8-week CPAP therapy. The questionnaire was completed in the presence of a trained medical professional who offered guidance when necessary.

The exclusion criteria were prior CPAP therapy, central sleep apnea, use of supplemental oxygen, non-OSA primary sleep disorder, major surgery or acute medical conditions in the prior 30 days, prior cardiovascular events, psychological disturbances, alcohol dependence, or other chronic diseases except metabolic syndrome. 

All patients signed a written informed consent for inclusion. The study was conducted in accordance with the Declaration of Helsinki [34], and the protocol was approved by the Ethics Committee of the Grigore T. Popa University of Medicine and Pharmacy in Iași (ethical approval code 1183/17.01.2018).

### 2.2. Study Design 

After OSA diagnosis, the patients were admitted to the Cardiovascular Rehabilitation Clinic of the Rehabilitation Hospital in Iași, Romania. Subjects underwent standard clinical examination and biological panel, ambulatory blood pressure monitoring (ABPM), and Epworth questionnaire. All patients were informed of the need for daily CPAP use and the importance of a healthy lifestyle (diet and exercise); no change was made to their current drug regimen. OSA patients received standard CPAP therapy with DreamStation Auto CPAP (Philips Respironics, Murrysville, PA, USA), REMstar Auto C-Flex CPAP (Philips Respironics, Murrysville, PA, USA), or AirSense 10 AutoSet CPAP (ResMed, San Diego, CA, USA). OSA patients were reevaluated in the same clinic, using the same procedures, 8 weeks after initiating CPAP therapy. After assessing CPAP adherence (at the 8-week follow-up), we divided our initial study population into two subgroups: adherent and nonadherent patients. Adherence was defined as a device usage time ≥ 4 h/night, while nonadherence was defined as a CPAP usage time < 4 h/night [35]. 

In the a priori calculation of the sample size and according to previous results on the effects of 8-week CPAP therapy on the arterial stiffness [12], we estimated that at least eight subjects were required for each subgroup to detect a mean absolute maximal improvement difference in Aix of 6.4%, after treatment, at a significance level α of 5%, β cut-off of 20% and statistical power of 80%. A previous meta-analysis reported an average adherence rate to CPAP of 83% (with a variation from 40 to 100%) [18]. As such, we recruited 39 patients to obtain at least 13 patients for each group.

### 2.3. Measurements

#### 2.3.1. Body Measurements 

All measurements were performed three times. Height and weight were assessed without shoes and with light clothing in the morning. Body mass index (BMI) was calculated as weight (kg)/height (m^2^). Waist circumference (WC) was measured at the end of a normal expiration, horizontally at the top of the right iliac crest, ensuring that the tape was snug, without compressing the patients’ skin.

#### 2.3.2. Smoking Status

Smoking status was classified as current smoker, former smoker, and never smoker, according to the National Health Interview Survey (NHIS) definition [36].

#### 2.3.3. CPAP Adherence

CPAP adherence data (device usage, hours per night at the prescribed pressure) was recorded by the machine and downloaded using the appropriate software: Encore Pro 2 v.2.17 (Philips Respironics, Murrysville, PA, USA), EncoreBasic v.2.1 (Philips Respironics, Murrysville, PA, USA), or ResScan v.6.0 (ResMed, San Diego, CA, USA). Adherence was defined by the time of CPAP use (≥4 h/night).

#### 2.3.4. ABPM 

The ABPM monitoring was performed with the DMS-300 ABP device (DM Software, Stateline, NV, USA) and was interpreted by an experienced cardiologist. The frequency of daytime (6:00–22:00) and nighttime (22:00–6:00) measurements was set at 30 and 60 min, respectively. The recording was considered satisfactory if it included at least 70% of the expected measurements. The first ABPM was performed before the initiation of CPAP. The second ABPM was performed after 8 weeks, with a full night of controlled CPAP use at home (data regarding accumulated CPAP use/56 days obtained from the device smart card).

#### 2.3.5. Holter-ECG

The Holter-ECG monitoring was performed using the three-channel DMS-300 4A Cardioscan (DM Software Stateline, NV, USA) device. The seven electrodes were positioned according to the Standard B pattern, following the manufacturer’s instructions [37]. The duration of the recording was 24 h. All recordings were manually interpreted by an experienced cardiologist. The first Holter-ECG was performed before the initiation of CPAP. The second Holter-ECG monitoring was performed after 8 weeks, with a full night of controlled CPAP use.

#### 2.3.6. Assessment of Arterial Function 

All patients underwent arterial stiffness assessment, before and after 8-week CPAP therapy, with the Arteriograph (TensioMed, Budapest, Hungary), using an appropriate cuff size, according to the patient’s arm circumference [38]. The 2 month re-evaluation of arterial stiffness was performed after a night of controlled CPAP use. The examination was conducted by a single operator, from 9:00 to 10:00 in accordance with the manufacturer’s instructions [38], in a quiet, temperature-controlled environment, after at least a 10 min rest period. The patient was not allowed to speak or move during the examination. Alcohol, caffeine, and smoking were not permitted 10 h prior to the examination. Central (aortic) systolic blood pressure (aoSBP), central (aortic) pulse pressure (aoPP), aortic augmentation index (aoAix), and aortic pulse wave velocity (aoPWV) were measured.

### 2.4. Statistical Analysis

The results were expressed as mean ± standard deviation or median (25th and 75th percentiles) for continuous variables and as percentages (%) for categorical variables. Statistical analysis was performed in SPSS v 20.0, using paired Student’s t-test and the Mann–Whitney U test for comparisons between groups for parametric and non-parametric variables, respectively. A linear mixed model using BMI as a covariate was performed for arterial stiffness parameters before and after CPAP. A potential relationship between variables was evaluated using the Pearson correlation coefficient. A *p*-value < 0.05 was considered the threshold for statistical significance.

## 3. Results

### 3.1. All Patients 

**Figure 1:**
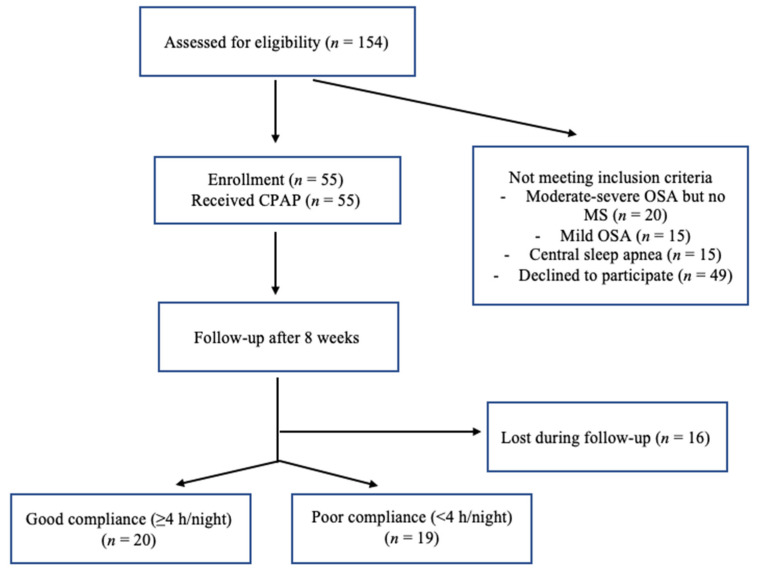
Flowchart diagram of patients examined in the sleep unit between January and December 2018; OSA: obstructive sleep apnea; MS: metabolic syndrome; CPAP: continuous positive airway therapy.

Of the 154 patients referred to our sleep unit between January and December 2018, 55 met the inclusion criteria. Of the remaining subjects, 20 had OSA but no MS, and 16 patients were lost during follow-up (Figure 1). 

**Figure 2:**
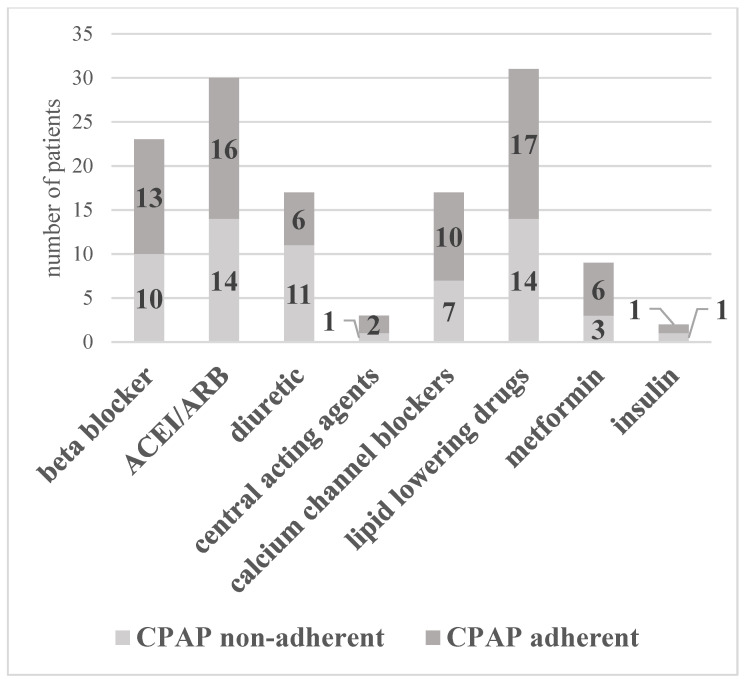
The use of anti-hypertensive, anti-diabetic, and lipid-lowering medications in the two subgroups, CPAP non-adherent and CPAP adherent. ACEI: angiotensin-converting enzyme inhibitors; ARB: angiotensin receptor blockers; CPAP: continuous positive airway pressure.

Our final study group included 39 patients (29 males and 10 females) with moderate–severe OSA and MS. The demographic, anthropometric, biochemical, and cardiorespiratory polygraphy characteristics are reported in Table 1. The average CPAP use in our entire study population (39 patients) was 4.0 ± 1.0 h/night. Average CPAP use was 2.3 ± 1.0 h/night and 6.1 ± 1.2 h/night in the non-adherent and adherent subgroups, respectively. The use of anti-hypertensive, anti-diabetic, and lipid-lowering medications were balanced across the two subgroups (Figure 2). 

### 3.2. Comparison between Groups

After 8-week CPAP therapy, we observed a statistically significant decrease in almost all arterial stiffness parameters (aoSBP, aoAix, and aoPWV) only in the CPAP adherent subgroup (Table 2). Both subgroups exhibited minor, but statistically significant changes in weight, BMI, and WC; however, this was not the case with BP and HR values, where no significant changes were observed (Table 2). 

After adjusting for BMI (linear mixed method), changes in aoPWV, aoAix and aoSBP remained significant in the adherent subgroup, but not among non-adherent patients, as follows: *p* = 0.004, 0.032 and 0.050 for aoPWV in all subjects, adherent and non-adherent patients, respectively; *p* = 0.024, 0.006 and 0.688 for aoSBP in all subjects, adherent and non-adherent patients, respectively; and *p* = 0.058, 0.011 and 0.468 for aoAix in all subjects, adherent and non-adherent patients, respectively. The change in aoPP was not significant in any of the analyzed subgroups after adjusting for BMI (linear mixed model).

**Figure 3:**
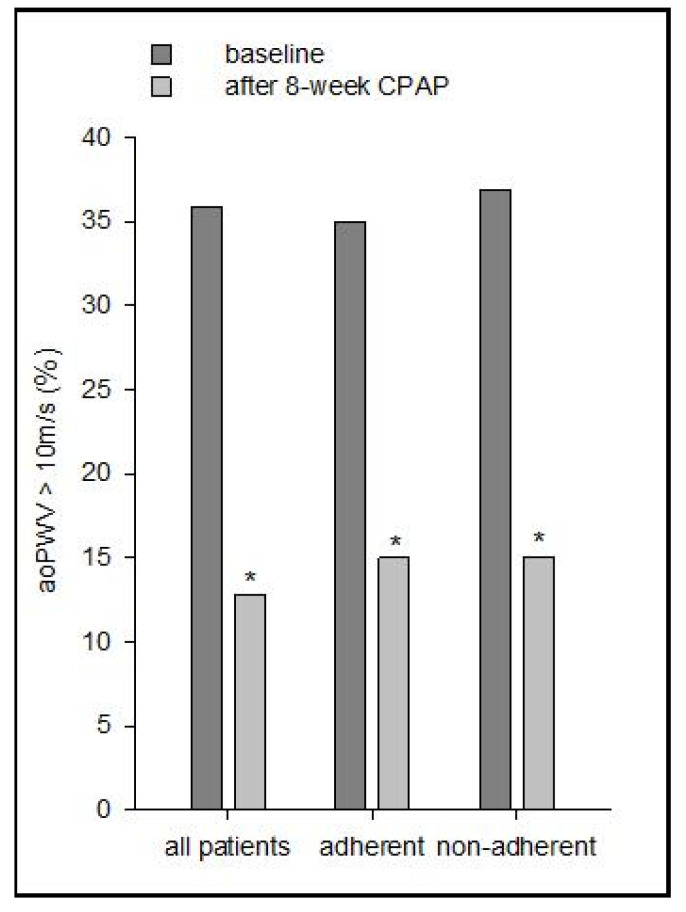
The prevalence of patients with aoPWV > 10 m/s at baseline and after 8-week CPAP therapy in the study population. aoPWV: aortic pulse wave velocity; CPAP: continuous positive airway pressure; *: *p* < 0.00001 vs. baseline.

The prevalence of patients with aoPWV > 10 m/s decreased from 35.89% (baseline) to 12.82% (after CPAP), *p* < 0.00001. A similar trend (*p* < 0.00001) was observed in the adherent and non-adherent subgroups (35% to 15% and 36.84% to 15%, respectively) (Figure 3).

### 3.3. Correlations between Arterial Stiffness and OSA Parameters 

**Figure 4:**
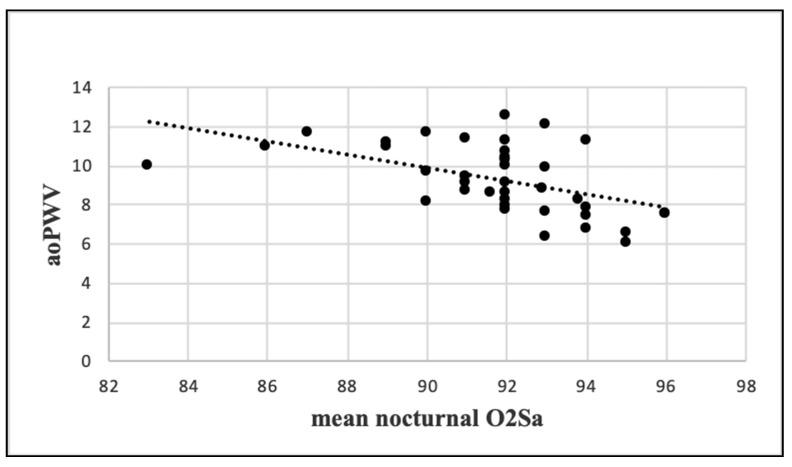
Correlation between aoPWV and mean nocturnal O2Sa. aoPWV: aortic pulse wave velocity; O_2_Sa: oxygen saturation.

Mean nocturnal O_2_Sa was significantly correlated with weight, BMI, and WC as well as exhibited a strong indirect correlation with aoPWV (r = −0.507, *p* = 0.0009) (Figure 4).

AHI was significantly correlated with the other OSA severity parameters, but not with age (*p* = 0.10) or arterial stiffness variables (Table 3). 

## 4. Discussion

Our study showed that short-term CPAP therapy significantly improved the arterial stiffness parameters in patients with moderate–severe OSA and MS; however, the benefit is greatly influenced by CPAP adherence, remaining statistically significant only in patients who used the device for >4 h/night. CPAP adherence in our study (51.28%) was significantly lower than that in previous reports [18,39], but similar to that observed by Dorkova et al. [40] in patients with severe OSA and MS. The poor CPAP adherence could be explained by the relatively low average ESS score observed in our study group [41], as well as by the presence of MS per se [42]. 

Determination using SphygmoCor (AtCor Medical, Sydney, Australia) or Complior (Artech Medical, Pantin, France) is the gold-standard for arterial stiffness evaluation [5]. However, cfPWV measurement is associated with a risk of carotid plaque rupture in the elderly and requires exposure of the groin area [5], thus limiting its routine clinical use. On the other hand, the advantages of Arteriograph (TensioMed, Budapest, Hungary) include the higher reproducibility of parameters, due to the simple and time-effective methodology [5]. Although it has been suggested that the Arteriograph (TensioMed, Budapest, Hungary) actually measures axillo-brachial stiffness (a parameter closely correlated with aoPWV) [43], the results we obtained with the Arteriograph (TensioMed, Budapest, Hungary) are similar to those obtained by standard devices used for arterial stiffness assessment [5,7,8]. Furthermore, a previous RCT found no difference between PWV measurements using SphygmoCor (AtCor Medical, Sydney, Australia) and Arteriograph (TensioMed, Budapest, Hungary) [44]. AoPWV measured using Complior (Artech Medical, Pantin, France) is significantly higher than that obtained using SphygmoCor (AtCor Medical, Sydney, Australia) or Arteriograph (TensioMed, Budapest, Hungary), mostly due to the different techniques used to measure pulse wave travel distance [44]. In order to avoid unnecessary bias due to the jugulum-pubic symphysis range, we used the first measured value for both Arteriograph (TensioMed, Budapest, Hungary) evaluations. 

The mean aoPWV in our study group was only 9.26 ± 1.73 m/s, and 35.89% of our patients had a baseline PWV value > 10 m/s. Short-term CPAP was associated with a decrease in the prevalence of patients with PWV > 10 m/s (Δ = −23.07%, *p* < 0.00001). OSA patients who associate hypertension or metabolic syndrome present the highest degree of arterial stiffening [11]. The initial threshold for PWV of 12 m/s proposed by the 2016 European guidelines on cardiovascular disease prevention [45] has been revised by the more recent 2018 guidelines for the management of arterial hypertension [4], in which a carotid-femoral PWV (cfPWV) > 10 m/s is considered a marker of asymptomatic hypertension-mediated organ damage (HMOD). 

The arterial stiffness parameters were not significantly different between our moderate and severe OSA subgroups (data not shown). Similarly, Protogerou et al. [46] did not find statistically significant differences concerning PWV and Aix between patients suffering from moderate, severe, and very severe OSA in the presence of cardiovascular comorbidities. Most studies support an independent, dose-response association between OSA and elevated arterial stiffness parameters [11,17]. In accordance with two recent meta-analyses [47,48], AHI was not correlated with the degree of arterial stiffening. However, contrary to the results of Joyeux-Faure et al. [48], in our analysis aoPWV was strongly correlated with mean nocturnal O_2_Sa, which was also associated with weight, BMI, and WC. Our results are in line with the ”hypoxemic burden” theory in OSA patients, in which an integrated symptom–comorbidity approach aims to replace AHI as the central parameter in the OSA treatment algorithm [41].

Despite the lack of a significant correlation between arterial stiffness and AHI, we found that short-term CPAP therapy significantly improved AoPWV by 0.66 m/s in our CPAP adherent subgroup, which is in accordance with a recent meta-analysis that showed that CPAP decreases arterial stiffness by 0.65 m/s in hypertensive patients [19]. Buchner et al. [49] also observed significant improvements in both Aix and PWV values (SphygmoCor) after 6 months of CPAP; this was only among the 49 effectively treated OSA patients, thus emphasizing the importance of CPAP adherence. Contrary to our results, in the study by Buchner et al. [49], the decrease in central SBP after CPAP among OSA adherent patients was not statistically significant. Differences in anti-hypertensive regimens between our study groups could explain this inconsistency.

CPAP reduces PWV (Complior, Artech Medical, Pantin, France) in patients with severe OSA, even in the absence of associated cardiovascular disease [20]. Interestingly, the PWV changes were correlated with improvements in C reactive protein serum concentrations [20], isolating OSA as an independent risk factor for both atherosclerosis and systemic inflammation. On the other hand, Jones et al. [14], in a double-blind placebo-controlled trial, reported that 12-week CPAP therapy did not significantly influence Aix or PWV in a group of 43 OSA patients without associated cardiometabolic comorbidities. However, the study population of Jones et al. [14] had significantly lower average AHI and BMI values compared with our study group (31 events/h and 29.9 kg/m^2^, respectively) and a baseline PWV of only 7.6 m/s (SphygmoCor, AtCor Medical, Sydney, Australia).

Medium-term CPAP use (6 months) was associated with a reduction in nighttime BP and arterial stiffness in patients with coexisting cardiovascular disease and OSA in a study by Picard et al. [50], but had no impact on arterial stiffness in patients with moderate–severe OSA and resistant HBP in another report by Cardoso et al. [51]. Kohler et al. [24] reported significant changes in Aix and mean arterial BP after only 4 weeks of therapeutic CPAP. One other study [25] evaluated CPAP-induced arterial stiffness changes in OSA patients with MS and reported significant improvements after 12 weeks of therapeutic CPAP [25].

Arterial stiffness is a composite measure of vascular health and an independent predictor of cardiovascular events [18]. Each 1 m/s increase in PWV is associated with a 15% increase in both cardiovascular and all-cause mortality [52]. As such, even a mild decrease in PWV can be clinically relevant, especially in patients with MS who are at increased cardiovascular risk. Recent studies have questioned whether arterial stiffness assessment using an oscillometric method (Mobil-O-Graph) can be used to predict cardiovascular outcome, since the measured PWV is highly dependent on patient’s age and SBP [53,54]. Although similar limitations could be attributed to the Arteriograph (TensioMed, Budapest, Hungary) device, aoPWV and aoSBP improvement occurred in the absence of statistically significant BP or HR variations (the most important confounding factors for PWV [29]), and remained significant after BMI adjustment. The mild decrease in aoPWV can be explained by the rather low baseline aoPWV (“pathological” values of >10 m/s [4] in only 35% of patients; Figure 1), by the short follow-up duration (8 weeks) and by the low initial ESS score [41]. CPAP seems to be more effective in improving BP in symptomatic OSA patients (excessive sleepiness) with associated cardiovascular comorbidities, including MS. Although aoSBP improved in CPAP adherent patients (Δ = −11.4 mmHg), average 24 h BP remained unaffected, suggesting that excessive sleepiness could be a more important predictor for positive CPAP effect on BP than the presence of MS. 

Interestingly, it was previously shown [26] that while PWV decreases over the first 6 months, it gradually increases during the following 18 months, without exceeding the baseline value. While this can be explained by the direct effect of aging and OSA on arterial stiffening, CPAP adherence and changes in chronic medication are important confounding factors and should be considered in the interpretation of these findings [23]. 

Despite being markedly dependent on cardiac characteristics and vascular tone (which are, in turn, altered in the presence of obesity in MS), Aix is sometimes used as a substitute parameter for arterial stiffness assessment [48]. A previous randomized trial reported a significant reduction in Aix after only one month of CPAP [24] and another study [12] reported that 8-week CPAP therapy improved morning (but not evening) Aix (Δ = 6.49%). Despite the identical therapy duration, the authors reported no improvement concerning PWV values, possibly due to preexisting irreversible OSA-related vascular damage [12]. Another explanation for the lack of significant PWV reduction is the relatively low prevalence of hypertension (63.9%). Furthermore, the study population of Paz y Mar et al. [12] included a high percentage of African Americans (45%) and females (47.3%) but had a significantly lower average AHI (19.3 events/h). 

Smoking status is an important confounder in arterial stiffness assessment [55]. Smoking is not permitted 10 h prior to Arteriograph (TensioMed, Budapest, Hungary) measurement, which could bias BP and arterial stiffness parameters in heavy smokers. We limited this confounding factor by scheduling an early Arteriograph (TensioMed, Budapest, Hungary) measurement (9.00 a.m.–10.00 a.m.).

Whether CPAP impacts on anthropometric parameters is controversial [56,57]. Our study group exhibited mild weight loss after 8 weeks of CPAP. All subjects received standard advice regarding diet and exercise as part of a healthy lifestyle; therefore weight-loss should not be attributed to CPAP only. Adherence to lifestyle changes was not the purpose of this study and was not quantified. Similar to other studies of short or medium-term CPAP therapy on arterial stiffness [20,24,49,51], we performed only a baseline evaluation of OSA severity.

To the best of our knowledge, our study is the first to use the Arteriograph (TensioMed, Budapest, Hungary) device to analyze the effect of CPAP on arterial stiffness in OSA patients with MS. Although the use of oschillometry to evaluate arterial stiffness has been recently criticized, the gold-standard evaluation of cfPWV is difficult to implement in clinical practice, as it is time-consuming, operator-dependent and subject to significant errors regarding the anatomical estimation of arterial length [53]. The impact of positive airway therapy on PWV and Aix is influenced by treatment duration and patient characteristics, i.e., daytime sleepiness, smoking status, medication, cardiovascular comorbidities, apnea severity and OSA duration before diagnosis [55]. Anti-hypertensive drugs are effective in reducing arterial stiffness [58], with significant variations between classes and within the same class [59]. However, the impact of these drugs on arterial stiffness parameters is partly (or mostly) attributable to their ability to reduce SBP values [58], which remained unchanged in our study group. The use of lipid-lowering and anti-diabetic drugs was relatively balanced between the two study groups. Subgroup analysis regarding the effect of each class of medicine on arterial stiffness parameters measured with the Arteriograph (TensioMed, Budapest, Hungary) was irrelevant due to the small number of patients, but should be addressed in future research.

Our analysis shows that the beneficial effects of CPAP on arterial stiffness are apparent only in adherent patients and occur in the early stages of CPAP therapy, in the absence of significant changes in mean BP or HR values. The oscillometric evaluation of arterial stiffness can be easily performed in daily practice and can be used as a surrogate marker of CPAP adherence, which could motivate the patient to use the device accordingly. As OSA and metabolic syndrome are cumulative cardiovascular risk factors, special attention should be given to patient education regarding optimal CPAP use.

## 5. Conclusions

Short-term (8 weeks) CPAP significantly reduced values for the aortic pulse wave velocity (aoPWV: Δ = −0.66 m/s, *p* = 0.03), aortic augmentation index (aoAix: Δ = −8.25%, *p* = 0.01) and central systolic blood pressure (Δ = −11.4 mmHg, *p* = 0.009) only in the CPAP adherent subgroup of OSA patients with MS. The Arteriograph (TensioMed, Budapest, Hungary) device is a noninvasive and time-effective way to assess arterial stiffness in patients with moderate–severe OSA and MS, yielding an in-depth analysis of individual cardiovascular risk.

## Figures and Tables

**Table 1:** Baseline demographic, anthropometric, biochemical, and cardiorespiratory polygraphy characteristics in the study population.

	All Patients (*n* = 39)	Adherent (*n* = 20)	Non-Adherent (*n* = 19)	*p*-Value ^#^
Age (years)	57 ± 9	60 ± 7	55 ± 10	0.080
Smoking status				
Current smoker (%)	12.8%	5%	21.1%	0.133
Former smoker (%)	56.4%	55%	57.9%	0.857
Never smoker	30.8%	40%	21.1%	0.200
Weight (kg)	101 ± 17	97 ± 17	105 ± 16	0.143
BMI (kg/m^2^)	33.8 ± 4.7	32.9 ± 4.8	34.7 ± 4.8	0.224
WC (cm)	114 ± 10	112 ± 10	116 ± 10	0.181
Blood tests				
Fasting blood glucose (mg/dL)	112.31 ± 19.84	111.77 ± 13.4	112.89 ± 25.31	0.863
HDL-cholesterol (mg/dL)	50.58 ± 12.87	52.80 ± 14.76	48.23 ± 10.41	0.274
TG (mg/dL)	167.44 ± 86.09	154.95 ± 98.88	180.60 ± 70.50	0.359
OSA parameters				
AHI (events/h)	39.7 ± 19.5	35.9 ± 15.9	45.4 ± 22.7	0.142
DI (events/h)	38.6 ± 18.4	35.6 ± 15.1	41.7 ± 21.4	0.312
Mean nocturnal O_2_Sa (%)	91.8 ± 2.6	91.8 ± 2.5	91.8 ± 2.7	0.907
ESS (points)	6.2 ± 3.9	6.9 ± 3.9	5.4 ± 3.9	0.245
CPAP pressure (cmH_2_0)	10.9 ± 2.3	11.5 ± 2.1	10.4 ± 2.5	0.159

Data are presented as mean ± SD. BMI: body mass index; WC: waist circumference; HDL: high-density lipoproteins; TG: triglycerides; OSA: obstructive sleep apnea; AHI: apnea–hypopnea index; DI: desaturation index; O_2_Sa: oxygen saturation; ESS: Epworth sleepiness score; CPAP: continuous positive airway pressure; ^#^: comparison between adherent and non-adherent subgroups.

**Table 2:** Impact of 8-week CPAP therapy on arterial stiffness parameters in our study group.

	All Patients (*n* = 39)			Adherent (*n* = 20)			Non-Adherent (*n* = 19)		
	Baseline	After 8-Week CPAP	*p*-Value	Baseline	After 8-Week CPAP	*p*-Value	Baseline	After 8-Week CPAP	*p*-Value
Age (years)	57 ± 9			60 ± 7			55 ± 10		
Weight (kg)	101 ± 17	98 ± 16	0.007	97 ± 17	95 ± 16	0.002	105 ± 16	102 ± 16	0.016
BMI (kg/m2)	33.7 ± 4.7	33.0 ± 4.6	0.0001	32.9 ± 4.8	32.3 ± 4.5	0.002	34.7 ± 4.8	33.8 ± 4.8	0.018
WC (cm)	114 ± 10	111 ± 11	<0.0001	112 ± 10	109 ± 10	0.000	116 ± 10	112 ± 12	0.002
ESS (points)	6.2 ± 3.9	3.1 ± 2.7	0.0001	6.9 ± 3.9	3.2 ± 2.5	0.0007	5.4 ± 3.9	3.1 ± 3.1	0.049
ABPM (mmHg)									
Mean SBP/24 h	129 ± 14	130 ± 13	0.597	129 ± 16	132 ± 9	0.372	129 ± 13	129 ± 15	0.965
Mean DBP/24 h	76 ± 8	75 ± 8	0.372	74 ± 7	75 ± 5	0.846	78 ± 8	75 ± 10	0.268
Mean daytime SBP	131 ± 16	133 ± 13	0.527	130 ± 19	134 ± 10	0.390	132 ± 13	132 ± 15	0.974
Mean daytime DBP	78 ± 9	77 ± 8	0.859	75 ± 10	77 ± 5	0.304	81 ± 8	78 ± 10	0.294
Mean nighttime SBP	122 ± 15	123 ± 14	0.877	125 ± 15	125 ± 11	0.936	120 ± 15	121 ± 17	0.796
Mean nighttime SBP	71 ± 10	68 ± 9	0.243	71 ± 8	69 ± 7	0.326	71 ± 12	68 ± 11	0.459
Holter ECG monitoring									
Mean HR/24 h (bpm)	71 ± 10	70 ± 9	0.641	70 ± 12	70 ± 10	0.526	70 ± 10	71 ± 8	0.175
Arterial stiffness									
aoSBP (mmHg)	122 ± 19	115 ± 13	0.029	125 ± 22	114 ± 15	0.009	118 ± 15	116 ± 13	0.696
aoPP (mmHg)	43.9 ± 11.9	41.9 ± 10.2	0.248	46.4 ± 13.4	42.4 ± 9.8	0.090	41.2 ± 9.9	41.6 ± 10.9	0.864
aoAix (%)	28.4 ± 15.2	24.7 ± 15.1	0.061	32.6 ± 16.2	24.3 ± 15.7	0.013	24.0 ± 13.1	25.2 ± 14.9	0.481
aoPWV (m/s)	9.3 ± 1.7	8.5 ± 1.4	0.004	9.2 ± 1.8	8.5 ± 1.3	0.036	9.3 ± 1.7	8.6 ± 1.6	0.057

Data are presented as mean ± SD. BMI: body mass index; WC: waist circumference; ESS: Epworth sleepiness score; SBP: systolic blood pressure; DBP: diastolic blood pressure; HR: heart rate; aoSBP: aortic systolic blood pressure; aoPP: central (aortic) pulse pressure; aoAix: aortic augmentation index; aoPWV: aortic pulse wave velocity.

**Table 3:** Correlations between OSA parameters with demographic and arterial stiffness parameters (*n* = 39).

	AHI r	*p*-Value	Mean Nocturnal O_2_Sa r	*p*-Value
AHI	-	-	−0.353	**0.027**
Mean nocturnal O_2_Sa (%)	−0.353	0.027	−	-
Desaturation index (events/h)	0.964	<0.0000001	−0.390	**0.013**
Age	−0.009	0.956	−0.101	0.537
Weight	0.257	0.112	−0.402	**0.010**
BMI	0.294	0.069	−0.469	**0.002**
WC	0.310	0.054	−0.568	**0.0001**
ESS	−0.088	0.598	−0.248	0.133
aoSBP (mmHg)	0.026	0.872	0.015	0.923
aoPP (mmHg)	−0.075	0.646	0.082	0.616
aoAix (%)	−0.235	0.149	0.201	0.218
aoPWV (m/s)	0.264	0.103	−0.507	**0.0009**

AHI: apnea–hypopnea index; O_2_Sa: oxygen saturation; BMI: body mass index; WC: waist circumference; ESS: Epworth sleepiness score; aoSBP: aortic systolic blood pressure; aoPP: aortic pulse pressure; aoAix: aortic augmentation index; aoPWV: aortic pulse wave velocity. Values in bold indicate statistically significant results.
